# Image guided focused ultrasound delivery of macromolecular drugs in tumours

**DOI:** 10.1186/2050-5736-3-S1-P75

**Published:** 2015-06-30

**Authors:** Maya Thanou, Mike Wright, Miguell Centelles, Wladyslaw Gedroyc

**Affiliations:** 1 King’s College London, London, United Kingdom; 2 Imperial College London, London, United Kingdom

## Background/introduction

Drug delivery using focused ultrasound (focal drug delivery) has attracted significant interest during the last few years. Focused Ultrasound (FUS) can induce local tissue hyperthermia, increasing blood flow and vascular permeability, which then enhances the uptake of therapeutics by target tissues. Although a number of studies have focused on nanoparticles (e.g. thermosensitive liposomes) little work has been done on the effects of FUS on the uptake of macromolecular drugs into tumours. In this study we aim to understand the effect of FUS-induced hyperthermia on tumour vascular permeability, as measured by the increased uptake of labelled macromolecules in the tumour. Using optical imaging we monitor the labelled macromolecules tumour localisation in real time in both short (minutes to hours) and long (weeks) term.

## Methods

Albumin (Murine Serum MSA), IgG, and Trastuzumab were labelled using a NIRF (Near Infrared Fluorescent probe; XL750) and a coupling protocol developed in house. The NIRF labelled macromolecules were purified using a PD-10 column and were characterised by HPLC and SDS-PAGE (both unheated and after heating to 42°C for 7 min). SCID mice were used to form tumours using IGROV-1 (ovarian cancer cells) injected s.c. to both flanks. When tumours reached suitable size (~6 mm diameter) animals were injected (i.v. tail vein) with the labelled antibodies. FUS applications were performed post injection, on the right tumour using an Ultrasound Therapy Imaging Probe System (TIPS, Phillips).

FUS insonations induced a localised temperature increase which was monitored using fine-wire thermocouples inserted above and below the tumour body. Temperature was kept at 42±0.3°C for 2-5 min. Multispectral optical imaging was used to assess the accumulation of labelled macromolecules in tumours over time with or without FUS-induced hyperthermia.

## Results and conclusions

For all three macromolecules tested *in vivo*, FUS induced hyperthermia enhanced significantly their uptake by the tumours. Figure shows the effect of FUS induced hyperthermia on murine serum albumin tumour uptake. FUS treatments induced immediate increase on macromolecular drug uptake by tumours. The macromolecules remained in the tumour for several days (with an enhanced signal for about a week). Trastuzumab appeared to persist in the tumours for a longer period probably due to its specificity for Her-2 receptors. The effect of this rapid and tumour localised macromolecular drug concentration can lead to an improvement of the therapeutic effect, this still needs to be investigated. Image guided antibody drug delivery using FUS can become a useful imaging and therapeutic tool.

**Figure 1:**
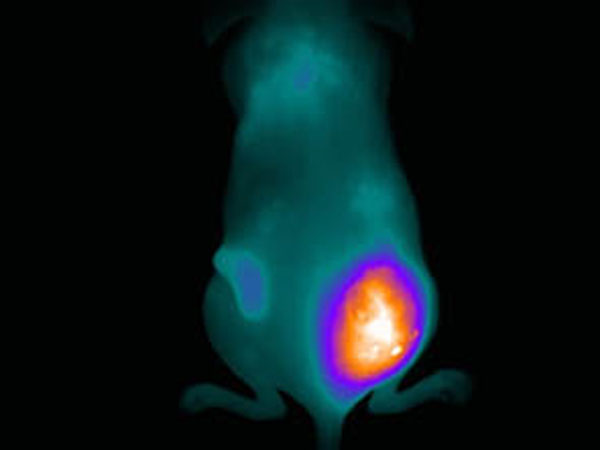
Optical imaging of NIRF –labelled albumin at 5 h post injection. Right tumour was treated with FUS to induce hyperthermia (post injection).

